# On best practices in the development of bioinformatics software

**DOI:** 10.3389/fgene.2014.00199

**Published:** 2014-07-02

**Authors:** Felipe da Veiga Leprevost, Valmir C. Barbosa, Eduardo L. Francisco, Yasset Perez-Riverol, Paulo C. Carvalho

**Affiliations:** ^1^Laboratory for Proteomics and Protein Engineering, Carlos Chagas Institute - FiocruzCuritiba, Brazil; ^2^Hexabio BioinformaticsCuritiba, Brazil; ^3^Systems Engineering and Computer Science Program, COPPE, Federal University of Rio de JaneiroRio de Janeiro, Brazil; ^4^Proteomics Services, European Molecular Biology Laboratory, European Bioinformatics InstituteCambridge, UK

**Keywords:** bioinformatics, best practices, source control, test, repository

## 1. Introduction

Bioinformatics is one of the major areas of study in modern biology. Medium- and large-scale quantitative biology studies have created a demand for professionals with proficiency in multiple disciplines, including computer science and statistical inference besides biology. Bioinformatics has now become a cornerstone in biology, and yet the formal training of new professionals (Perez-Riverol et al., 2013; Via et al., 2013), the availability of good services for data deposition, and the development of new standards and software coding rules (Sandve et al., 2013; Seemann, 2013) are still major concerns. Good programming practices range from documentation and code readability through design patterns and testing (Via et al., 2013; Wilson et al., 2014). Here, we highlight some points for best practices and raise important issues to be discussed by the community.

## 2. Source-code availability to reviewers

It is debated among researchers whether source codes should be made available to reviewers, as doing so could allow for a more complete review and evaluation of the manuscript’s results. It could also ultimately enable reviewers to demand quality and clarity in the same way as from manuscripts originating from laboratory experiments, in which a bad PCR or a Western-Blot without controls may lead to wrong interpretations of the results (Ince et al., 2012). In the case of software, a clear indication that best practices were not followed can bespeak carelessness and therefore indirectly signal that something may be wrong. It is our opinion that reviewing the source code from submitted papers should be possible if desired, though publishers would obviously have to search for even more specialized reviewers for the task. The review process does not necessarily need to be done at the code level but can be accomplished by evaluating the structure of the project, availability of test units, and functional tests. By organizing and providing tests with different case scenarios the authors can easily demonstrate how the software works and how it behaves in different occasions. The possibility of executing the code (without having to go deeply into it) and of looking into how particular issues are handled in the code is important at all stages of the work (both pre- and post-publication). Further inspection by the scientific community will eventually lead to the same advantages we see in open-source projects like the Linux kernel (Torvalds, 2014b) or the protocols used in the Internet. Bugs can be spotted and improvements suggested by the community. This is especially important because, as science is an ever changing enterprise, always adapting and growing, the opportunity is given for the software to evolve along with the field.

## 3. Software indexing and availability

A topic that we should address as a community is the possibility of indexing software with a solution like the well-known DOI system. An example of such an initiative is the combined work of the Mozilla Science Lab (Mozilla Foundation, 2014), GitHub (GitHub, 2014), and Figshare (Figshare, 2014). This would enable researchers and practitioners to easily keep track of different software versions, thereby facilitating access and deployment (Summers, 2014). Currently, it is common for bioinformatics software to be hosted by university or even personal or laboratory websites. Although they are convenient and provide users with quick access to the material in question, such solutions are also the source of a major problem in bioinformatics, namely the discontinuation of software availability. An ideal solution to this problem would be a central hosting repository where each version could be archived and made available. This would also help when old versions became necessary for old, third-party workflows. Another important aspect is the ability to prevent the deletion of previous versions of a project, which would also help prevent other projects from ceasing to exist after a certain time or being abandoned.

## 4. Documenting the source code

Software documentation can be categorized into two groups, one targeted at software developers, the other at the end users. The former is usually found in the source code, or is linked to it, and is used to explain the particularities of the code itself, which is important especially for software updating and customization. The latter typically uses nontechnical language and is aimed at aiding the user in the process of software installation and execution. Without proper code documentation the process of resolving a bug or including new developers in the team becomes a very complicated task. Users likewise need to have access to the documentation explaining its usage, which must include all directives for installation under different operating systems (when such is the case) and for the handling of parameters and input data prior to a run. It is also important to note that we need proper documentation for biologists, as they will be the ones installing and using the programs. With easy-to-follow guidelines and instructions for non-programmers, it is possible to improve software usability.

## 5. Source-code management

During a software’s life cycle, a varying number of developers can be involved with its production and different versions of it can be created. One of the main goals of having source-code management is to have all these aspects automatically taken care of through the building of a historical registry of development. Solutions such as Git allow the simultaneous collaboration with several projects while greatly simplifying each maintainer’s tasks of tracking and resolving bugs, handling feature requests, and launching upgrades (Torvalds, 2014a). This also helps to promote the collaborative aspect of software development since anyone can join an ongoing project and provide patches.

## 6. Test libraries, sample data, and dataset repositories

A test library is a series of scripts designed to test a given piece of software. It is meant to aid in quickly determining whether the software’s main modules are working as expected. Ideally, all functions of the code should be thus tested, but sometimes this is not possible because of the size or complexity of the project. What is fundamental to test, though, is whether the main logic and operations are working correctly whatever the running environment happens to be. Normally a test library is shipped together with the software and the tests are executed before installation to certify that the main features are working on the machine at hand. Another important aspect of any scientific software is that sample data be provided along with it, in a manner similar to that in which supplementary files are provided together with a manuscript. Through “real-world” examples, users can verify what to expect of the various analyses. Such examples also allow for comparisons with other datasets (Perez-Riverol et al., 2014).

## 7. The advantages of the open-source development

There are several advantages to making a software project open source (Perez-Riverol et al., 2014). In computer science, projects are usually classified into two major categories: open source and proprietary. Being open source means making the code freely available, a simple gesture that can have powerful implications for user projects, especially those that are science-related. One of the greatest advantages of an open-source program is that it is possible to see and understand all functionalities and every calculation it does, thus ensuring full transparency. The same cannot be said of proprietary software, in which case users are required, essentially, to have faith in the product’s developer/seller and become unable to criticize or properly know how results are obtained. In general, open source means a greater tendency toward reliability, as anyone can peruse the source code and eventually spot some bug. As such, an open-source project is continually reviewed by the community. When someone spots an error and then corrects it, a patch can be generated and sent to the code maintainer. One of the key aspects of having an open-source project is to provide clarity about how results are generated and can be reproduced (Prli and Procter, 2012).

## 8. Final considerations

During the development phase of a software project, adopting best practices in programming involves investing time and effort to better structure ideas as both the code and the documentation are written. Although such investment may at times seem cumbersome, in the long run it benefits both developers and users, and is therefore valuable. In a related vein, another crucial issue is trustworthiness: from the perspective of the scientists using it, a software tool abiding by good practices can provide more confidence as their own projects are developed, which in turn is a key aspect of any work based on data analysis. All of this point in the direction of the software having more quality, since ultimately, quality depends on programming practices. The more quality a software has, the longer it will live and the more people will use it (Altschul et al., 2013). In this regard, a noteworthy initiative is the GMOD Galaxy, an open and integrated workflow system which allows the sharing of customized analyses (Giardine, 2005). Other examples of softwares following the best practices listed above are Tophat (Trapnell et al., 2009), Bowtie (Langmead et al., 2009), and the BioPerl project (Stajich, 2002).

### Conflict of interest statement

The authors declare that the research was conducted in the absence of any commercial or financial relationships that could be construed as a potential conflict of interest.
